# Cross-Linking and Corneal Imaging Advances

**DOI:** 10.1155/2015/306439

**Published:** 2015-04-08

**Authors:** A. John Kanellopoulos, Ronald R. Krueger, George Asimellis

**Affiliations:** ^1^Laservision.gr Clinical and Research Eye Institute, 17 Tsocha Street, 115 21 Athens, Greece; ^2^Department of Ophthalmology, NYU Medical School, New York, NY, USA; ^3^Case Western Reserve University, Cleveland, OH, USA

## 1. Introduction

Corneal cross-linking, a technique employing UV-A illumination and a photomediator to induce corneal rigidity, is a widely recognized procedure for the stabilization or even possibly reversal of corneal ectasia progression in patients with keratoconus and post-LASIK ectasia. A rapidly growing number of clinical reports suggest a consistent stabilizing effect of cross-linking along with a variable improvement in corneal shape and visual function in some patients. In the past ten years there has been a continuous effort into understanding, ensuring safety and efficacy, and further expanding its applications, as well as exploring modifications aiming to optimize the technique. Research in the field of CXL is highly dynamic; techniques, concepts, and indications are constantly evolving. Recent advances in corneal cross-linking include applications for infections treatment that do not respond to topical medications; accelerated, high-fluence applications; prophylactic application in refractive surgery; modified beam profiles for selective treatments; fully customized induction of refractive changes in nonectatic eyes. We welcome in this special issue several papers on this subject covering topics such as the issue of epithelial removal with hypotonic riboflavin solution, as well as a contralateral study on this subject; study investigating rate of corneal collagen cross-linking redo, investigating risk factors and safety, including a study investigating the profile of microbial keratitis following CXL; long-term investigation of safety and visual outcome of Visian toric ICL implantation after CXL in keratoconus; long-term investigation of accelerated CXL in paediatric patients; biomechanical effects investigation of the correlation between tomographic and biomechanical severity of keratoconic corneas; and a novel application of intraoperative optical coherence tomography in CXL.

Keratoconus is considered an unpredictably progressive eye disease that “softens” the cornea. The progressive thinning and “bulging” of the cornea may distort or even significantly reduce vision. In advanced cases, one or more corneal transplant procedures and possibly additional eye surgeries may be required for visual rehabilitation. As it mainly affects younger people, it has severe consequences in their quality of life and their ability to contribute to the active workforce during their most productive years. In our experience within our ophthalmology center in Greece, through extensive studies conducted the last 10 years, we have found that in unpublished data possibly more than 1 out of 35 patients display some form of keratoconus in modern cornea diagnostics, compared to 1 out of 1,000–2,000 reported in Northern Europe and the United States. In addition, we have noted a higher degree of familial correlation of keratoconus reaching 90% topographic or tomographic suspicion in one of the two parents of a known young adult with keratoconus, a marked difference compared to the 10% genetic correlation that has been previously reported.

Over the last decade a new treatment, collagen cross-linking (CXL), has been introduced. In this treatment, vitamin B2 and ultraviolet light (UV-A) are applied to the cornea in a short procedure that “stiffens” the cornea and stops disease progression.

## 2. Current Treatment Options for Keratoconus Management

Keratoconus progression was traditionally observed. Visual rehabilitation was managed with spectacle correction and/or soft contact lenses, until irregular astigmatism necessitated application of rigid gas permeable (RGB) contact lenses.

In cases when this was not possible or there was RGP intolerance (estimated up to 21% of cases [1]), traditionally, a penetrating keratoplasty (PK) in which the patient's cornea is discarded and replaced with a fresh donor cornea was employed. This procedure is associated with significant morbidity [2], as usually it takes about a week for the patient to return to normal everyday life and months, if not years, before that eye can be adequately visually rehabilitated. It is noted that, despite the use of this drastic procedure, visual rehabilitation may still necessitate additional repair and/or refractive procedures in order to reduce the very common irregular astigmatism and high postoperative anisometropia associated with penetrating keratoplasty.

Even in cases where PK generally achieved acceptable visual outcomes, long-term graft survival in keratoconic eyes declined rapidly after the second decade because the endothelial cells of the donor cornea tend to be slowly rejected by the host. Primary graft survival rates have been reported to 50% at 20 years [3], falling even further with repeat grafts.

An alternative to PK is deep anterior lamellar keratoplasty (DALK) which does not have the disadvantage of short lifespan and associated complications. In DALK this risk is possibly lower as the endothelial cell layer of the host is preserved: a median graft survival of 49 years for DALK versus 17 years for PK has been reported. It is noted, however, that DALK techniques are technically challenging.

Other introduced treatment options for keratoconus are the insertion of intracorneal ring segments (ICRS). These inserts appear to significantly shift the shape of the cornea and may provide significant visual rehabilitation. Although, in clinical use for several years, there is no unison assessment of their stability and safety, we have reported along with other clinicians a number of significant short- and long-term complications associated with the ICRS.

Collagen cross-linking, on the other hand, has proven that it can effectively arrest the progression of keratoconus and corneal ectasia. The standard, epithelium-off Dresden protocol has been proven to be effective in arresting keratoconus progression.

Despite substantiated safety, we have reported, along with other clinicians, a range of complications associated with CXL. In addition to the standard CXL, other protocol variations introduced include alternative levels and amounts of energy, pulsing, oxygen supplementation, riboflavin solution concentrations, and route of administration within the cornea of the riboflavin solution. The underlying premise of these alternatives is that delivering a similar effect over a shorter period of time will not compromise safety in comparison with the standard protocol.

Our team has contributed many of the evolutionary steps of the initially introduced CXL technique:

1. higher fluence,
2. use of dextran-free riboflavin solution,
3. combination of CXL with topography-guided excimer normalization of ectatic corneas (the Athens Protocol),
4. prophylactic CXL in routine myopic and hyperopic LASIK,
5. in situ CXL through a femtosecond laser created corneal pocket,
6. photorefractive CXL.

Specifically, we have introduced the concept of accelerated, high-fluence collagen cross-linking (CXL) in post-LASIK ectasia, as well as the utilization of prophylactic CXL in routine LASIK, and in situ, femtosecond laser-assisted treatment of corneal ectasia, in attempting corneal deturgescence in bullous keratopathy, and as a prophylactic intervention adjuvant to Boston keratoprosthesis surgery.

## 3. The Need for Comparative Evaluation of CXL Protocols

Over the last ten years CXL has evolved to be a valid treatment for the arrest of the progression of keratoconus. Since the original Dresden protocol (3 mW/cm^2^ for 30 minutes), several treatment CXL protocol variations have been introduced, most of them by our team [4]. These, however, have not been fully compared as far as their correlating effect. These variations involve higher fluence, such as the use of 6, 10, 18, and 30 mW/cm^2^, and correspondingly shorter UV-A exposure time, aiming to deliver the same (5.4 J/cm^2^) or more total amount of energy, and presumably adequate stiffening effect [5]. Besides the original CXL protocol parameters evaluated more extensively, all newer-introduced CXL protocols have not been evaluated and correlated as extensively either clinically or ex vivo. In order to correlate the efficacy of the standard to newer CXL protocols the following prospective studies must be conducted, both clinically and ex vivo with the following parameters:

- ectasia stabilization (topographic and anterior elevation stability and/or improvement),
- safety in regard to visual acuity loss, corneal clarity, corneal inflammation, and endothelial cell loss,
- biomechanical/biochemical response parameters.

To the best of our knowledge, so far no direct and thorough comparative study of these CXL protocols has been conducted. The lack of CXL-techniques comparison is a noteworthy shortcoming. In a recent example, in a smaller-scale precursory prospective randomised trial carried by our team, contralateral eyes of 21 patients with progressive keratoconus were randomised to either conventional or high-fluence CXL (7 mW/cm^2^ for 15 min).

Several assessment modalities for the evaluation of CXL efficacy exist. They include ex vivo biomechanical (tensile strength), biochemical (enzymatic digestion) [6], and in vivo methods, for example, via OCT imaging demarcation line [7], corneal hysteresis (CH), and corneal resistance factor (CRF). CH is considered indications of corneal viscous damping, reflecting the capacity of corneal tissue to absorb and dissipate energy; CRF is considered an indicator of the overall corneal resistance.

The latter may be evaluated by dynamic tonometry (visualization of fast deformation of the cornea), employing the Corvis ST (Oculus Optikgerate GmbH, Wetzlar, Germany) and the Ocular Response Analyzer (Reichert, Buffalo, NY). The Corvis ST is a functional in vivo corneal biomechanics analyzer employing a noncontact tonometer and enabling recording the corneal reaction to an air impulse. An incorporated high-speed Scheimpflug camera (4,330 frames/sec) records still frames of the oscillating cornea. The device enables assessment of corneal biomechanics for various applications of refractive surgery, keratoconus screening, and cross-linking assessment.

Several studies have evaluated the reduction in corneal biomechanical strength following refractive surgeries such as LASIK. However, there is inconclusive evidence in the peer-review literature on the specificity of these techniques in the evaluation of the effect of corneal cross-linking [8].

Ex vivo corneal biomechanical evaluation may be conducted with biaxial stress-strain measurements. The BioTester 5000 (Cell Scale, Waterloo, Ontario, Canada) is a specifically developed biomaterials biaxial strength analyzer applicable to ex vivo corneal rigidity (Young's modulus) measurements within a temperature-controlled media bath. Two high-performance actuators (two per axis) are capable of *μ*m positional resolution for accurate test motion, with inline overload-protected load cell on each axis. The device captures and graphically displays live time, force, and synchronized video images for results analysis and verification. Data are easily exported to standard spreadsheet programs. Future promising diagnostics may include devices that are based on phonon spectroscopy as demonstrated already in studies on the Brillouin-based investigative devices.

*A. John Kanellopoulos* *Ronald R. Krueger* *George Asimellis*
